# Supplementary material for: Enrichment of Activated Fibroblasts as a Potential Biomarker for a Non-Durable Response to Anti-Tumor Necrosis Factor Therapy in Patients with Crohn’s Disease
Source: Int J Mol Sci. 2023 Sep 30;24(19):14799. doi: 10.3390/ijms241914799 (PMC10573580; doi:10.3390/ijms241914799)
Supplement: Supplementary file 1 [file ijms-24-14799-s001.zip › Supplementary Figure S1.pdf]

DEG	Non-inflamed vs Uninflamed(CD)	DEG	Non-inflamed_1 vs Inflamed_2
*qcFtest	2,368	*qcFtest	3,066
**Up**(non-inflamed)	**1,594**	**Up**(non-inflamed_2)	**1,373**

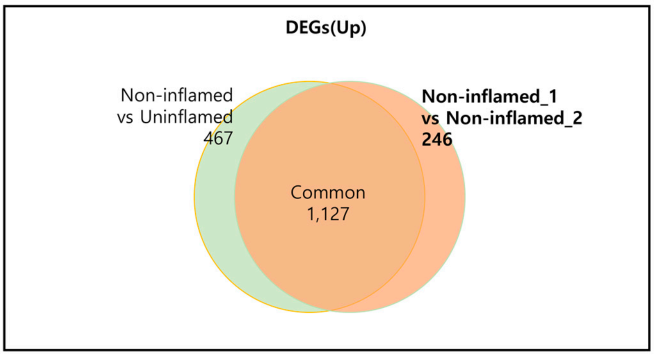

*qcFtest = p-value < 0.05 & Abs(LogFC) > 1
